# Enhancing comfort of resident physicians treating adults with intellectual and developmental disabilities by facilitating meaningful interactions

**DOI:** 10.3389/fmed.2024.1264958

**Published:** 2024-05-21

**Authors:** Jessica Solomon Sanders, Kathryn Williams, Darcy Thompson, Hannah F. Shapiro

**Affiliations:** ^1^School of Medicine, Department of Pediatrics, Section of Developmental Pediatrics, University of Colorado, Denver, CO, United States; ^2^Boston Children’s Hospital, Institutional Centers for Clinical and Translational Research, Biostatistics and Research Design Center, Boston, MA, United States; ^3^School of Medicine, Department of Pediatrics, Section of Nutrition, University of Colorado, Denver, CO, United States; ^4^Department of Neurology, UCSF Benioff Children’s Hospital, University of California, San Francisco, San Francisco, CA, United States

**Keywords:** medical education, developmental disability, residency, intellectual disability, graduate medical education

## Abstract

**Background:**

Many physicians feel uncomfortable caring for patients with intellectual and developmental disabilities (IDD). While some residency training programs include lecture content on IDD, few provide structured experiences with individuals with IDD. One strategy for improving comfort is “contact theory:” increasing interactions with “dissimilar” people can lead to decreased negative attitudes toward that population.

**Objective:**

Evaluate the impact of an interactive session on resident physicians’ comfort with adults with IDD.

**Methods:**

Small groups of resident physicians and artists with IDD collaborated on art projects during the noon conference. A prospective pre-post-intervention survey, including the validated Interaction with Disabled Persons Scale (IDP), evaluated residents’ comfort with patients with IDD before and after the session.

**Results:**

53 residents completed both pre- and post-conference surveys. Mean IDP scores decreased from 78.7 (10.9) to 75.8 (9.5; *p* < 0.01), indicating decreasing discomfort. The mean level of comfort interacting with individuals with IDD increased from uncomfortable 3.6 (1.2), before the intervention, to comfortable 4.4 (1.2) after the intervention (*p* = <0.01). The mean level of comfort treating individuals with IDD increased from uncomfortable 3.5 (1.1) to comfortable 4.1 (1.3) after the intervention (*p* < 0.01).

**Discussion:**

Providing resident physicians with real-life connections with people with IDD was associated with increased comfort. If statistically significant improvements occurred after one session, future studies should evaluate if additional experiences with people with IDD could have more substantial, lasting impacts on future doctors’ comfort with and willingness to care for patients with IDD.

## Introduction

Increasing numbers of adults with intellectual and developmental disabilities (IDD) are living into adulthood in the community (1–3). Often physicians are not trained in caring for this patient population (4) and feel uncomfortable accepting these patients into their clinical practice (5). In addition to explicit negative attitudes, people with disabilities often face implicit bias, which refers to unconscious social sentiments. One systematic review found that people with disabilities were implicitly stereotyped as incompetent, cold, or “child-like” (6). Provider discomfort is one of the main barriers individuals with IDD face to accessing age-appropriate, high-quality healthcare (7–9).

Current IDD-related education in residency programs is not well described. While there have been increasing efforts to include IDD-related education in medical training, including the publication of Core Competencies on Disability for Healthcare Education (10), this type of education is not widely required in graduate or undergraduate medical education (11–13). A survey of Internal Medicine primary care residency programs revealed that most program directors felt that internists and their own residents were inadequately prepared to offer equitable care to individuals with disabilities, and only a minority offered disability-focused residency curricula (14).

The Accreditation Council for Graduate Medical Education mandates common program requirements for competency in professionalism. Among five competencies residents are expected to demonstrate includes, “respect and responsiveness to diverse patient populations, including but not limited to diversity in gender, age, culture, race, religion, disabilities, national origin, socioeconomic status, and sexual orientation” (15).

One scoping review enumerated teaching strategies effective in educating medical students about disability including presentations, group sessions, lectures, workshops, presentations by guest lectures, online modules, and seminars (16). A survey of 14 medical schools found that most curricula that addressed disability competency had limited engagement with people with disabilities (17). While there have been pilot studies that suggest didactics and simulated patient experiences can help trainees feel more comfortable caring for individuals with disabilities, it is not known whether interacting with individuals with IDD in a nonclinical setting could improve physicians’ comfort with treating this population (18, 19).

Increasing physicians’ comfort with interacting with individuals with an IDD in a nonclinical setting may be one possible way to improve physicians’ comfort with treating this population. One well-established strategy for improving comfort with individuals with disabilities is based on contact theory: (20) increasing interactions with “dissimilar” people can lead to decreased negative attitudes toward that population (21). Whereas simple contact or exposure has proven not to be sufficient, meaningful interactions in which dissimilar groups engage in structured activities and have common goals have sustained the most impact. Contact theory proposes that there will be reduced prejudice four features of the contact situation are present: equal status between the groups; common goals; intergroup cooperation; and the support of authorities (22). While contact theory was originally formulated to address racial prejudice, it has been researched and effective across many other groups and situations ranging from people of different ages, physical abilities, and mental illnesses (22). We developed an education program, rooted in contact theory, aiming to increase trainees’ comfort caring for adults with IDD by providing opportunities for meaningful contact.

We hypothesized that residents would report higher comfort levels interacting with and treating patients with IDD after the interactive intervention. We also aimed to explore the intervention’s impact on secondary outcomes of barriers to treating patients with IDD and identification of what interventions would improve resident comfort with patients with IDD.

## Methods

### Study design

We used a prospective pre-post intervention design to assess resident physicians’ comfort with adults with IDD using survey questionnaires. This was not part of a larger study.

### Participants and settings

All residents in four residency training programs, two adult neurology and two internal medicine, were invited to participate during their hour-long residency noon conference September to December (10).

Adults with IDD from Gateway Arts, a non-profit organization that employs artists with IDD, were selected by their staff to participate in the sessions.

### Intervention

The intervention, informed by contact theory, aimed to facilitate a quality interaction between residents and adults with IDD. Designed through an academic-community partnership with a community non-profit organization that employs artists with IDD (Gateway Arts), the artists with IDD sat with resident physicians at tables during their noon conference didactic sessions. For every 5 residents at a table, there was at least one artist with IDD from Gateway Arts. Most tables had 2–3 artists and 5–10 residents. To begin the sessions, the artists with IDD sat at tables, and leaders from the residency programs directed residents to sit evenly at each table as the residents filed into the room for the session. There was a brief introduction to Gateway Arts and their organization’s mission and operations in the community. Staff from the community organization provided groups with personalized “conversation starter cards” to facilitate dialog and enable resident physicians to learn about the lives, personalities, and strengths of the artists.

To enhance further engagement, artists and resident physicians engaged in a shared art project, creating the opportunity to work together toward a shared goal. Shared art projects were chosen by the Gateway Arts artists and staff based on the skills of each artist with disabilities who participated. Examples of shared art projects included collage-making from magazines, painting pumpkins, and bead work. The reason for the cooperative project being an art project was to level the hierarchical structure of the participants. By highlighting the strengths of the participants with IDD, the typical doctor-patient hierarchy was lessened since the residents were learning skills from the artists with IDD.

This intervention was piloted for 3 years prior to the study period. The 3 years of pilot programming consisted of continuous improvement and change cycles based on feedback from staff and artists from our community partner as well as resident participants and residency leadership. The intervention was developed in collaboration with a community non-profit called Gateway Arts that supports and employs adults in the community. In developing the interactive programming, we included individuals with lived experience, including those with IDD and their supporters. The participants with IDD were compensated for their time.

### Procedures

We collected surveys 1 week before and immediately after the interactive conferences using both electronic (REDCap) and paper forms. The survey was developed in consultation with the Institutional Centers for Clinical and Translational Research at Boston Children’s Hospital. Participants received gift cards for completing each survey. Approximately 75% of the residents who attended the conferences, resulting in 53 residents completing both pre- and post-conference surveys.

### IRB statement

The Boston Children’s Hospital Institutional Review Board (IRB) determined that this survey-based study was exempt from human subjects’ review.

### Measures

#### Provider comfort measure

Provider comfort was assessed through two survey questions asking participants to rate their comfort interacting with and treating individuals with IDD. Response options were on a scale of 1–6, with no neutral point: 1 “very uncomfortable,” 2 “somewhat uncomfortable,” 3 “a little uncomfortable,” 4 “a little comfortable,” 5 “somewhat comfortable,” and 6 “very comfortable.”

#### Interactions with disabled person scale

Primary outcome measures assessed provider discomfort and comfort with individuals with IDD. We evaluated provider discomfort with the validated 20-item Interaction with Disabled Persons Scale (IDP) (23), which was “designed to measure emotions, motivations and reactions that underlie negative attitudes associated with discomfort that some people experience in actual or anticipated social interaction with a person with a disability.” A higher total score on the IDP indicated more discomfort in interactions.

#### Perception of barriers to treating patients with IDD

Secondary outcomes measured resident perceptions of barriers affecting one’s own ability to treat patients with IDD, interventions to improve resident comfort, and satisfaction with the intervention. We asked participants to rate the impact that 14 barriers had on their comfort treating patients with IDD. Residents rated each barrier 1 through 4: 1 “not at all,” 2 “a little,” 3 “somewhat,” and 4 “very much” a barrier.

#### Interventions to increase comfort treating patients with IDD

Residents also rated six intervention options on each intervention’s potential to increase comfort treating patients with IDD: More interactions with people with IDD; more didactic sessions/lectures about IDD; a dedicated rotation about care for patients with IDD; online resource with facts and practice guidelines about patients with IDD; having dedicated support staff (RNs, resource specialist, case coordinator, etc.) to navigate services for patients with IDD; having a doctor who specializes in caring for patients with IDD in my clinic/department to talk through cases of patients with IDD.

### Statistical analysis

Descriptive statistics included frequency counts and percentages for categoric variables and means for continuous variables. The non-parametric Wilcoxon signed-rank test was used to test pre-and post-intervention scale differences. This test was chosen because the differences were not normally distributed. The Fisher’s exact test was used to test differences between groups, pre-and post-intervention, for categorical measures. All tests were 2-sided and a *p*-value<0.05 was considered statistically significant. SAS (version 9.4, SAS Institute, Cary, NC) software was used.

For a *post-hoc* power analysis, we considered the results of the level of comfort interacting with individuals with IDD. There is 99% power to detect a different of 0.8 (4.4 vs. 3.6, sd = 1.2) using a two-sided, one-sample Wilcoxon Signed-Rank test α = 0.05, with a sample size of 45 (90% power with a sample size of 28).

## Results

53 residents completed both pre- and post-conference surveys. Participant characteristics, prior experiences with persons with IDD, and prior education on the topic are outlined in Table 1. Prior to the intervention, nearly all residents (98%, *n* = 52) reported previously treating a patient with IDD, but only 13% (*n* = 7) reported receiving formal education about IDD during their residency training. Supplementary Figure 1 details the types of prior experiences residents had with individuals with IDD.

**Table 1:** Characteristics of residents participating in intervention aiming to improve comfort with patients with IDD (*n* = 53).

Characteristics	N (%) or mean (SD)	
Gender		
Woman	27 (50.9)	
Man	25 (47.2)	
Other	1 (1.9)	
Age*	28.8 (2.2)	
Residency Year		
1	12 (22.6)	
2	16 (30.2)	
3	10 (18.9)	
4	13 (24.5)	
5	2 (3.8)	
Residency Specialty		
Internal Medicine	34 (64.2)	
Neurology	17 (32.1)	
Adult subspecialty track**	2 (3.8)	
Prior resident experiences with patients with IDD		
Prior experience	N	N (%)
Treated a patient with IDD	53	52 (98.1)
Received education dedicated to teaching about IDD	53	7 (13.2)
Prior experience interacting with people with IDD	53	
None		1 (1.9)
A little		39 (73.6)
Some		11 (20.8)
Much		2 (3.8)

SD, Standard Deviation. *N* = 52. Adult subspecialty track includes residents training in radiology, surgery/surgical subspecialty, and pathology.

Comfort levels differed before and after the intervention. From the Interactions with Disabled Persons (IDP) scale, the mean discomfort score prior to the interactive conference was 78.7 (Standard Deviation 10.9) compared to 75.8 (9.5) after the intervention (*p* < 0.01). The lower mean score following the intervention indicates lower levels of discomfort.

Before the interactive sessions, 42% (19/45) of residents reported they were comfortable interacting with individuals with IDD (scale level 4–6), and 71% (32/45) reported being “comfortable” after the interaction. Similarly, before the intervention, 44% (17/39) of residents reported their comfort in the “comfortable” range (scale level 4–6) pre-intervention and 64% (25/39) were “comfortable” post-intervention. Of the 45 participants who completed the comfort interacting scale before and after the intervention, 26 participants (58%) reported they were in the “uncomfortable” range (scale level 1–3) prior to the intervention. Of these 26 participants, 16 (62%) shifted to the “comfortable” range after the intervention. The remaining 10 participants (38%) stayed in the “uncomfortable” range. Of the 39 participants who completed the comfort treating scale before and after the intervention, 22 participants (56%) reported they were in the “uncomfortable” range (scale level 1–3) prior to the intervention. Of these 22 participants, 10 (45%) shifted to the “comfortable” range after the intervention. The remaining 12 participants (55%) stayed in the “uncomfortable” range.

**Figure 1:**
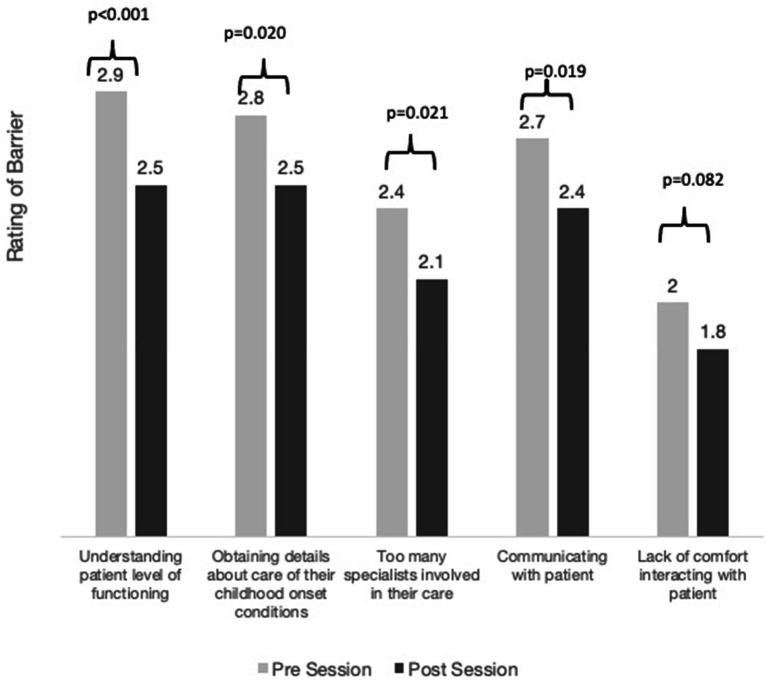
Perceived barriers before and after the interactive sessions. Residents rated each barrier 1 through 4: 1 “not at all,” 2 “a little,” 3 “somewhat,” and 4 “very much” a barrier.

Perceived barriers to treating a person with IDD also changed after the intervention. Following the intervention, residents reported that both difficulty understanding a patient’s level of functioning and difficulty communicating with a person with IDD were less of a barrier to their ability to treat a patient with IDD compared to prior to the intervention (*p* < 0.001, *p* < 0.05, respectively; Figure 1). Resident assessments of whether different interventions would increase their comfort caring for a patient with IDD did not change significantly from before to after the session (Supplementary Table 1). After the interactive session, however, 88.1% rated “more interactions” higher than they rated “more didactics,” vs. 64.3% before the session (*p* < 0.05). The three highest rated interventions were having dedicated support staff, having a doctor who specializes in IDD available to talk through cases, and having more interactions with persons with IDD.

95.2% of residents (*n* = 42) reported they would either definitely (61.9%, *n* = 26) or probably (33.3%, *n* = 14) recommend this session to other residents.

## Discussion and next steps

After the facilitated interactions, resident physicians reported increased comfort and decreased discomfort with individuals with IDD, a group with which many doctors have expressed fear, anxiety, or lack of confidence (5, 24–26). Meaningful contact between individuals with IDD and trainees was an educational strategy associated with improved physician comfort interacting with, and ultimately treating, a population of patients that so many doctors have dismissed as outside of their comfort zone (27).

Additionally, the sessions decreased perceived barriers in treating this patient population. Most compelling were: less difficulty understanding the patient’s level of functioning and less difficulty communicating with patients with IDD.

Most residents reported only having “a little” experience interacting with people with IDD, highlighting that many residents go through training without ample supportive experiences despite the high frequency in which they provide medical care for people with IDD. While there were varied prior experiences with people with IDD, the most common experience was taking care of a person with IDD on the inpatient service, further highlighting that many interactions with this population occur when these patients are at their “worst”: sick and hospitalized.

Notably, after one interactive session, residents reported decreased perceived barriers in communicating with and understanding patients with IDD. The current body of literature supports the need for increased training in communicating with people with IDD; (28) however, most educational programs currently in place are didactic in nature (7, 8). Interestingly, in our study, when presented with options of activities that could potentially increase resident comfort in providing medical care for patients with IDD, residents rated “more interactions with people with IDD” higher than they rated “more didactics.”

The effect size on the validated Interactions with Disabled Persons Scale was −2.9 in our study. This effect size is similar to what has been reported in the literature (29). Our study rooted in contact theory showed similar improvement with comfort with individuals with disabilities as did another study that included communication training plus direct contact with “tutors” with intellectual disability (29). Future work should investigate if combining communication training and a contact-theory-based intervention improves comfort even more.

There were limitations to our study. While the participants represented several residency programs, the small sample size still limits generalizability of the findings. Also, pre-post studies using self-report surveys are inherently subjective and we did not have a control group for comparison. Although the response rate of 75% is high for a survey-based study, it is possible that some response bias was introduced from who did participate. Although our single-item comfort measures of interacting with and treating patients with IDD were not validated in this initial study, we did include the Interactions with Disabled Persons (IDP) scale, a validated measure where lower scores indicate higher comfort. The results of both of our single-item comfort measures of interacting and treating patients with IDD showed that higher comfort score post-intervention correlated with a lower score post-intervention on the IDP scale (Spearman correlation: r_s_ = −0.54, *p* < 0.001 and r_s_ = −0.66, *p* < 0.001 respectively). We did not utilize subscales of the IDP for factor analysis; we utilized total IDP scores. Future studies should utilize the Discomfort subscale, as it is the most reliable subscale from the IDP (30).

### Future directions

Future work should build on this preliminary evaluation of an intervention rooted in contact theory and investigate the impact that longitudinal patient contact with patients with IDD has on resident perceived comfort treating this population. Additionally, future directions should include an evaluation of residents who receive didactic training in addition to opportunities to care for patients with IDD feel more comfortable treating patients with IDD. Didactic instruction plus active involvement in patient care during residency is the normative model that engenders the ability for doctors to comfortably care for patients without IDD; given that 87% of sampled residents did not receive IDD-related didactic training, it would be helpful to know whether the normative model, didactic training plus active involvement during residency, would also work for treatment of patients with an IDD.

Future studies could assess more objective measures regarding residents’ change in behavior toward this patient population. Additional studies are needed to assess the sustainability of this type of intervention and to better understand the optimal frequency of facilitated interactions. Also, the addition of a control group in future studies would strengthen the conclusions of further work.

Additionally, future work should evaluate the impact that improved physician-reported comfort has on the care that individuals with IDD receive. An important future direction for this research would be to measure whether patients with IDD themselves actually feel like they have received better care from physicians who report higher comfort levels with individuals with IDD following this intervention, relative to physicians who did not report higher comfort levels and/or did not undergo the intervention.

## Conclusion

Providing resident physicians with real-life connections and experiences with people with IDD led to increased comfort levels and decreased discomfort in caring for patients with IDD after one interactive session. Medical training programs should ensure that residents receive training on caring for individuals with IDD to the same depth that they receive training on caring for individuals without an IDD. We hope that incorporating meaningful interactions with patients with IDD into more residency curricula will not only improve resident comfort with this population but will improve patients with IDD’s access to the high-quality healthcare that all patients deserve.

## Data availability statement

The raw data supporting the conclusions of this article will be made available by the authors, without undue reservation.

## Ethics statement

Ethical approval was not required for the study involving humans in accordance with the local legislation and institutional requirements. Written informed consent to participate in this study was not required from the participants or the participants' legal guardians/next of kin in accordance with the national legislation and the institutional requirements.

## Author contributions

JS: Writing – original draft, Writing – review & editing. KW: Formal analysis, Methodology, Writing – review & editing. DT: Writing – review & editing. HS: Writing – original draft, Writing – review & editing.
