# Supplementary material for: Enhancing comfort of resident physicians treating adults with intellectual and developmental disabilities by facilitating meaningful interactions
Source: Front Med (Lausanne). 2024 May 21;11:1264958. doi: 10.3389/fmed.2024.1264958 (PMC11148428; doi:10.3389/fmed.2024.1264958)
Supplement: Supplementary file 1 [file Data_Sheet_1.docx]

**Supplementary Table 1**: Interventions that residents feel would increase their comfort caring for a patient with IDD

| Interventions | Pre-session  mean (SD) | Post-session mean (SD) | Difference of  mean (SD) | P-value |
| --- | --- | --- | --- | --- |
| Having dedicated support staff (RNs, resource specialist, case coordinator, etc.) to navigate services for patients with IDD n=42 | 3.8 (0.5) | 3.7 (0.5) | 0 (0.6) | ≥ .05 |
| Having a doctor who specializes in caring for patients with IDD in my clinic/department to talk through cases of patients with IDD n=42 | 3.4 (0.7) | 3.2 (0.7) | -0.1 (0.8) | ≥ .05 |
| More interactions with people with IDD n=42 | 3.0 (0.8) | 3.2 (0.6) | 0.2 (0.7) | ≥ .05 |
| More didactic sessions/lectures about IDD n=42 | 2.8 (0.8) | 2.7 (0.8) | 0 (0.8) | ≥ .05 |
| Online resource with facts and practice guidelines about patients with IDD n=42 | 2.6 (0.9) | 2.8 (0.7) | 0.2 (1.1) | ≥ .05 |
| A dedicated rotation about care for patients with IDD n=42 | 2.6 (1.0) | 2.7 (0.8) | 0.1 (0.7) | ≥ .05 |

Scale of 1-4: 1 = “not at all,” 2 = “a little,” 3 = “somewhat,” 4 = “very much.”

**Supplementary Figure 1**: Types of Resident Experiences with People with IDD (n=53)
